# Synergistic antibacterial and osteogenic activities of GO/GelMA hydrogel for regenerating periodontal bone defects

**DOI:** 10.3389/fbioe.2026.1744639

**Published:** 2026-04-23

**Authors:** Cheng Cheng, Lu Qiang, Guangna Yue

**Affiliations:** 1 Department of Stomatology, Beijing University of Chinese Medicine Third Affiliated Hospital, Beijing, China; 2 Department of Stomatology, Shanghai East Hospital, Tongji University School of Medicine, Shanghai, China

**Keywords:** antibacterial, GelMA, graphene oxide, osteogenesis, periodontitis

## Abstract

**Objective:**

This study aims to develop and assess graphene oxide (GO) and methacrylated gelatin (GelMA) composite hydrogels (GO/GelMA) for their combined antibacterial and osteogenic properties in the treatment of periodontitis-induced bone defects through comprehensive *in vitro* and *in vivo* experiments.

**Methods:**

GO was synthesized via a modified Hummers method and incorporated into GelMA to form composite hydrogels. The physical properties, including porosity, swelling behavior, and mechanical strength, were characterized. Antibacterial activity was evaluated against *Porphyromonas gingivalis* (*P. gingivalis*). The osteogenic potential was assessed by culturing rat bone marrow mesenchymal stem cells (BMSCs) on the hydrogels and analyzing cell proliferation and differentiation. A murine periodontitis model was used for *in vivo* validation.

**Results:**

The GO/GelMA hydrogels exhibited favorable physicochemical properties for bone tissue engineering. The incorporation of GO significantly enhanced the antibacterial performance, effectively inhibiting the *P. gingivalis* growth in a concentration-dependent manner. *In vitro* cell experiments demonstrated that while the 100 µg/mL GO/GelMA group showed the highest cell viability, the 200 µg/mL GO/GelMA group exhibited superior osteogenic differentiation. Correspondingly, *in vivo* results revealed that the 200 µg/mL GO/GelMA group achieved the most significant alveolar bone regeneration and the lowest levels of inflammatory factors.

**Conclusion:**

The GO/GelMA hydrogels, particularly at a GO concentration of 200 µg/mL, demonstrate excellent synergistic antibacterial and osteogenic properties, holding great promise for treating periodontitis-related bone defects.

## 1 Introduction

Periodontitis is a prevalent chronic inflammatory disease that primarily affects periodontal tissues, including alveolar bone, leading to tooth mobility and loss (Vaquette et al., 2018). Current therapeutic strategies focus on controlling inflammation, restoring periodontal health, and repairing bone defects. However, conventional treatments, such as scaling, root planing, and periodontal surgery, frequently fall short in achieving complete bone regeneration (Fleischer et al., 1989; Graziani et al., 2017).

With advances in tissue engineering and regenerative medicine, periodontal tissue regeneration has become a research focus. An ideal scaffold for periodontal regeneration should possess good biocompatibility, osteoconductivity, mechanical stability, and antibacterial activity to support regeneration while preventing infection (Vaquette et al., 2018). Hydrogels have emerged as promising candidates due to their structural similarity to the native extracellular matrix (ECM), which effectively mimics *in vitro* cell culture conditions and provides three-dimensional (3D) support for tissue formation (Herrera-Ruiz et al., 2022; Mamidi et al., 2023). Their tunable mechanical strength, excellent biocompatibility, and favorable bioactivity make them highly suitable for bone regeneration applications.

Among various hydrogel materials, gelatin methacryloyl (GelMA) stands out due to its low immunogenicity, ease of functionalization, and photocrosslinking capability (Kurian et al., 2023; Zhou et al., 2023; Huang et al., 2025). Its microstructure supports cell proliferation and differentiation, making it suitable for bone tissue engineering (Caldwell et al., 2019; Sun et al., 2025). For instance, digital light processing (DLP)-based fabrication of methacrylated silk fibroin (Sil-MA) micro-needles has demonstrated enhanced mechanical strength and transdermal delivery efficiency (Tong et al., 2025), illustrating the potential of photocrosslinked systems in biomedical applications.

Recently, carbon nanomaterials such as graphene oxide (GO) have been incorporated into hydrogels to enhance their mechanical and biological properties of scaffolds. GO exhibits a high surface area, antibacterial activity, and biocompatibility, making it a promising additive for bone regeneration (Zhao et al., 2010; Lee et al., 2011). The abundant functional groups on GO, such as carboxyl (-COOH) and hydroxyl (-OH) groups, can form chemical bonds with the hydrogel matrix, thereby enhancing the osteogenic differentiation of bone mesenchymal stem cells (BMSCs) (Qin et al., 2020; Pulingam et al., 2021). Furthermore, the π–π bonds in GO enable the adsorption of proteins and ions, which further promotes the proliferation and differentiation of stem cells (Purohit et al., 2019).

In this study, we developed an integrated therapeutic strategy for the complex microenvironment of periodontitis-induced bone defects. Rather than a simple physical blend, we designed a photocrosslinked GO/GelMA composite hydrogel with GO nanosheets uniformly dispersed and fixed within the GelMA network. This design seeks to achieve sustained functional release and address challenges such as nanoparticle aggregation and inconsistent bioavailability. By combining GO with GelMA, we integrated their advantages to develop a periodontal tissue regeneration material with both antibacterial and osteogenic properties. Specifically, we focused on optimizing the GO concentration to balance antibacterial efficacy and osteogenic differentiation, ultimately establishing a comprehensive strategy for periodontal bone regeneration.

## 2 Materials and methods

### 2.1 Synthesis and characterization of GelMA/GO

#### 2.1.1 Synthesis and characterization of GO

GO was synthesized using a modified Hummers method (Qin et al., 2020). Briefly, graphite powder (3 g) was mixed with sodium nitrate (40 mL) and concentrated sulfuric acid (230 mL) under ice bath conditions. Subsequently, 18 g of potassium permanganate was slowly added with continuous stirring for 1 h. Then, 3 mL of 30% hydrogen peroxide solution was added dropwise until the solution turned golden yellow. The product was obtained by centrifugation and dried under vacuum to obtain GO powder.

The surface morphology of GO was observed using scanning electron microscopy (SEM, Hitachi S-3400, Tokyo, Japan). Surface functional groups of GO were analyzed using Fourier-transform infrared spectroscopy (FTIR, Bruker TENSOR27, Billerica, MA, USA). The size and thickness of GO was characterized using atomic force microscopy (AFM, Bruker Dimension FastScan, USA).

#### 2.1.2 Synthesis and characterization of GelMA

**FIGURE 1:**
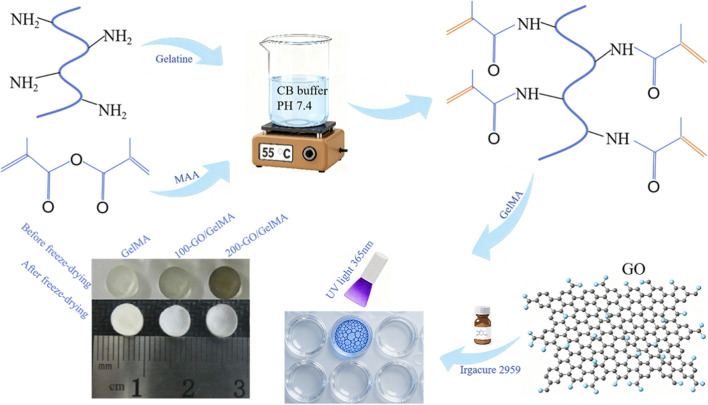
The preparation scheme of GelMA and GO/GelMA composite hydrogel.

Type A gelatin (10 g) was dissolved in 100 mL of CB buffer solution, heated to 55 °C and stirred at 600 rpm for 30 min. Subsequently, 0.8 mL of methacrylic anhydride (MAA) was slowly added, and the pH was adjusted to 7.4. The reaction proceeded at 50 °C for 1.5 h. After filtration and dialysis for 7 days, the product was lyophilized and stored at −20 °C. All materials were purchased from Sigma-Aldrich, USA. The synthesis scheme is shown in Figure 1.

The degree of substitution of GelMA was determined using H-NMR spectroscopy (JEOL Resonance ECZ 400S spectrometer). For analysis, 40 mg of gelatin and 40 mg of GelMA were separately dissolved in 1 mL of deuterium oxide (D_2_O). Spectra were acquired at 40 °C. The degree of substitution (DS) was calculated based on characteristic peak areas according to the literature (Mamaghani et al., 2018), using the following formula:

$$DS\;\left(\%\right)=\left(1-\frac{A-G\text{eltin}}{A-\text{GelMA}}\right)\times 100\%$$ (1)

where A-Gelatin was the area of methylene-lysine in the gelatin and A-GelMA was the area of GelMA methylene in the GelMA.

#### 2.1.3 Preparation and physical characterization of GO/GelMA hydrogels

10 wt% GelMA was dissolved in 1×phosphate-buffered saline (PBS), and 0, 100, and 200 µg/mL of GO and 0.2 wt% photoinitiator (Irgacure 2959, Sigma-Aldrich, USA) were added. The mixture (100 µL) was transferred to a 96-well plate and photocrosslinked under UV light (365 nm, 350 mW) for 1 min to form cylindrical hydrogels with a height of 3 mm and a diameter of 6 mm (GelMA group, 100-GO/GelMA group, and 200-GO/GelMA group). The samples were freeze-dried and stored at −20 °C. The morphology of the hydrogels was examined by SEM (Fei Nova Nanosem 450, USA).

Porosity of the hydrogels was determined as described previously (Farag et al., 2022). Briefly, a certain amount of anhydrous ethanol was measured, and the freeze-dried samples were completely immersed in the ethanol for 30 min. The mass of the hydrogel was measured before and after immersion. Porosity was calculated using the following formula:

$$Porosity\;\left(\%\right)=\frac{W2-W1}{\rho V}\times 100\%$$ (2)

where W1 and W2 are the masses of the hydrogel before and after immersion in ethanol, ρ is the density of anhydrous ethanol, and V is the volume of the freeze-dried hydrogel before immersion.

The swelling ratio was evaluated in PBS with at pH 7.4 and room temperature (Farag et al., 2022). The freeze-dried hydrogels were weighed and marked as W3. The hydrogels were then immersed in PBS for 24 h. After swelling, the hydrogels were taken out, and the excess surface moisture was gently blotted with filter paper. The hydrogels were weighed again and marked as W4. The swelling ratio was calculated using the following formula:

$$Swelling\;Ratio\;\left(\%\right)=\frac{W4-W3}{W3}\times 100\%$$ (3)

*In vitro* biodegradation rate was determined as reported (Saravanan et al., 2013; Farag et al., 2022). Hydrogels were weighed and marked as W5, then immersed in PBS buffer containing lysozyme (1,000 U/L) at 37 °C for 4 weeks. After incubation, the hydrogels were blotted dry and lyophilized. The mass of the lyophilized hydrogels was recorded as W6. The degradation rate was calculated using the following formula:

$$Degradation\;\left(\%\right)=\frac{W5-W6}{W5}\times 100\%$$ (4)

After soaking the GelMA, 100-GO/GelMA, and 200-GO/GelMA hydrogels in PBS for 24 h, the mechanical properties were tested using a mechanical testing system (HY-0230, Engyi, China) at a rate of 1 mm/min until the samples ruptured. The elastic modulus was calculated based on the resulting stress-strain curves (Zheng et al., 2023).

### 2.2 Antibacterial assay

The *in vitro* antibacterial performance was assessed using the agar plate method. Porphyromonas gingivalis (*P. gingivalis*, ATCC 33277) strain used in this study was obtained from the Microbiology Laboratory of Capital Medical University. The frozen *P. gingivalis* was resuspended in sterile brain heart infusion (BHI, Oxoid Ltd., UK) liquid medium and cultured anaerobically at 37 °C for 24 h. The bacterial solution was then diluted and evenly spread on blood agar plates. Single colonies with black pigmentation were selected and resuspended for further culture for 20–24 h.

The freeze-dried hydrogel discs were soaked in PBS to reach swelling equilibrium, sterilized under UV light for 30 min, and then immersed in bacterial suspensions (1 mL, 1.0 × 10^8^ CFU/mL). The suspensions were transferred to an anaerobic incubator and shaken at 160 rpm for 24 h. Subsequently, 100 µL of each suspension was spread on blood agar plates and incubated overnight. The optical density at 600 nm (OD600) was measured using a UV-Vis spectrophotometer (Epoch 2, Bio-Tek, USA). Bacterial survival rate was calculated using the following formula:

$$Bacterial\;Survival\;rate\;\left(\%\right)=\frac{\text{Nt}}{N0}\times 100\%$$ (5)

where Nt represents the number of colonies in the 100-GO/GelMA, and 200-GO/GelMA groups, and N0 represents the number of colonies in the GelMA group.

Additionally, the antibacterial performance of the hydrogels was further evaluated using live/dead bacterial staining. The bacteria treated with different hydrogels were co-stained with PI/SYTO9 (L-7012, Invitrogen, USA) for 20 min in the dark, followed by three washes with PBS. According to the manufacturer’s instructions, SYTO9 labels all bacterial cells green, and PI labels dead cells red. Fluorescence images were acquired using a confocal laser scanning microscope (CLSM, Leica, Germany).

### 2.3 *In Vitro* osteogenic evaluation

#### 2.3.1 Isolation and culture of BMSCs

BMSCs were isolated from the femurs and tibias of 4-week-old Sprague-Dawley rats as described (Qin et al., 2020). Cells were cultured in high-glucose Dulbecco’s modified Eagle’s medium (DMEM, HyClone, USA) supplemented with 10% fetal bovine serum (FBS, Gibco, USA) and 1% penicillin/streptomycin (HyClone, USA) at 37 °C with 5% CO_2_. BMSCs at passages 2%–4% and 80%–90% confluency were used for subsequent experiments.

#### 2.3.2 BMSC viability assay

GelMA, 100-GO/GelMA, and 200-GO/GelMA hydrogels were placed in 96-well plates. BMSCs were seeded onto the surfaces of the hydrogels at a density of 1 × 10^3^ cells/mL and cultured for 1, 3, and 7 days. Cell viability was assessed using the Cell Counting Kit-8 (CCK-8, AbD Serotec, Oxford, USA). Ten microliters of CCK-8 solution was added to each well, incubated for 2 h, and the absorbance at 450 nm was measured using a BioTek instrument. Each experiment was repeated three times. To visualize the cells on the hydrogel surfaces, live/dead staining (Molecular Probes, Eugene, USA) was performed after 3 days of culture.

#### 2.3.3 Alkaline phosphatase (ALP) and alizarin red S (ARS) staining

BMSCs cultured on hydrogels were induced with osteogenic medium for 2 (ALP) or 3 (ARS) weeks. ALP staining was performed using a commercial kit (Beyotime). For ARS, samples were incubated with 1% alizarin red solution (pH 4.2) for 10 min.

#### 2.3.4 Real-time quantitative PCR (RT-qPCR) analysis

After 14 days of osteogenic induction, total RNA was extracted using Trizol and quantified spectrophotometrically at 260 nm. cDNA was synthesized and RT-qPCR was performed using SYBR Green master mix and gene-specific primers (Supplementary Table S1). Glyceraldehyde-3-phosphate dehydrogenase (GAPDH) was used as the internal reference gene for normalization, and the fold change in gene expression was calculated using the2 ^−ΔΔCt^ method.

#### 2.3.5 Western blotting

The expression of osteogenic-related proteins was analyzed by Western blot at days 7 and 14. Total cell protein was extracted, separated by polyacrylamide gel electrophoresis, and transferred to a poly-(vinylidene fluoride) membrane (Millipore), and the expression levels of Runx-2 and OPN proteins were detected. GAPDH was used as the internal control.

#### 2.3.6 *In vivo* animal experimental

A total of 15 male C57BL/6 mice (6–8 weeks old) were used to induce periodontitis by ligation for 2 weeks. Briefly, mice were anesthetized by intraperitoneal injection of telazol (40 mg/kg) combined with dexmedetomidine (0.1 mg/kg). A silk thread was then placed around the mesial and distal surfaces of the upper molars on both sides of the maxilla using a size 4–0 suture. Then, these mice with periodontitis were randomly divided into three groups (N = 5 per group). GelMA group: mice treated using 50 μL GelMA hydrogel solution; 100-G0/GelMA group: mice treated using 50 μL 100-GO/GelMA hydrogel solution; and 200-GO/GelMA group: mice treated using 50 μL 200-GO/GelMA hydrogel solution. Finally, the hydrogels were cured by exposure to UV light (365 nm, 350 mW) for 1 min.

Following a treatment period of 2 weeks, mice were euthanized through a lethal dose of pentobarbital (150 mg/kg). The maxillae and gingival tissues were then removed and preserved in 4% PFA. The expression levels of inflammatory factors (IL-1β, IL-6 and TNF-α) in the gingival tissue were assessed using RT-qPCR. The sequences for all primers utilized are detailed in Supplementary Table S1. The distance from the alveolar bone crest to the cementoenamel junction (CEJ--ABC) of the affected teeth was observed and measured using a stereoscopic microscope (Sunny, Shanghai, China). Alveolar bone regeneration was evaluated by micro-CT (SkyScan 1,176, Bruker) and quantified using CTAn software for parameters including bone volume/tissue volume (BV/TV), trabecular number (Tb.N), thickness (Tb.Th), and separation (Tb.Sp).

### 2.4 Statistical analysis

Data were analyzed using SPSS 23.0 software. Normality and homogeneity of variance were tested. One-way ANOVA was used to analyze differences within groups, and t-tests were used to analyze differences between the control and experimental groups. A p-value <0.05 was considered statistically significant.

## 3 Results

### 3.1 Physical characterization of GO and GO/GelMA hydrogels

**FIGURE 2:**
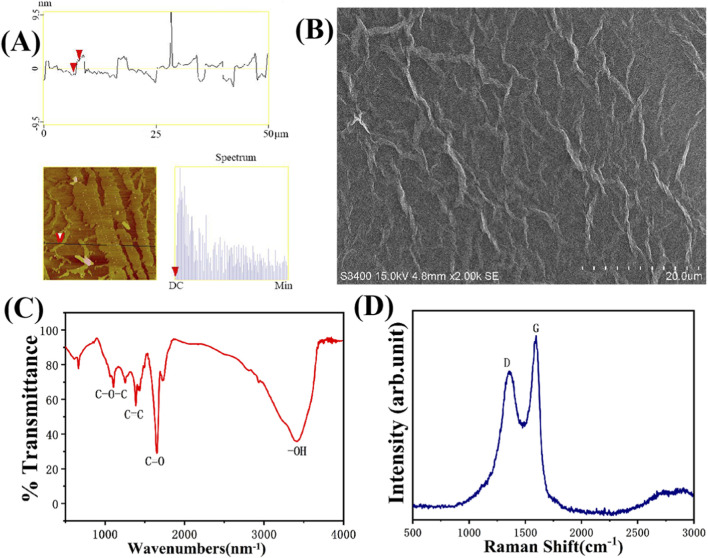
Rheological characterization of GO. **(A)** AFM images. Arrowheads denote the height profile of the selected GO nanosheet. **(B)** SEM image revealing the characteristic wrinkled morphology of GO nanosheets. **(C)** FTIR spectrum identifying characteristic functional groups, including C=O, C=C, and C–O–C. **(D)** Raman spectrum showing the distinctive D and G bands, indicating structural changes during oxidation.

AFM results (Figure 2A) showed that the thickness of GO was approximately 1.0 nm, with lateral dimensions reaching several hundred nanometers. SEM results (Figure 2B) revealed that GO nanosheets formed wrinkled structures due to van der Waals forces. FTIR results (Figure 2C) showed characteristic peaks at 1,630 cm^-1^, 1,380 cm^-1^, and 1,090 cm^-1^, corresponding to the stretching vibrations of C=O, C=C, and C–O–C groups, respectively, indicating that the sp^2^ carbon domains on GO nanosheets were occupied and that a large number of oxidized groups were present, resulting in good dispersibility in aqueous systems. Raman spectroscopy (Figure 2D) showed a sharp G peak at ≈1,588 cm^-1^ and a D peak at ≈1,350 cm^-1^, indicating structural changes during graphite oxidation.

**FIGURE 3:**
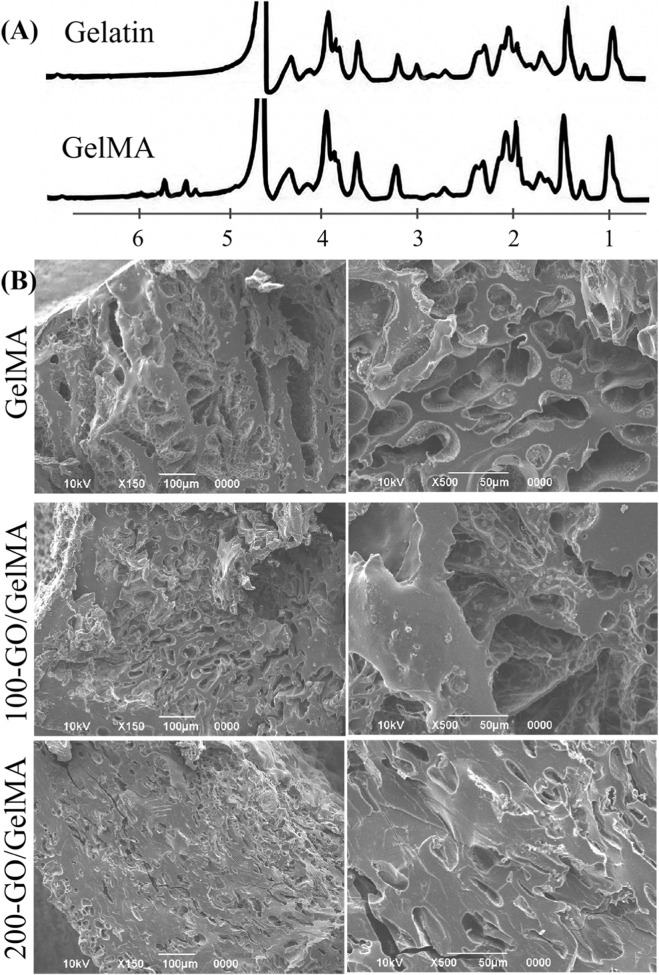
Characterization of GO/GelMA hydrogels. **(A)** H-NMR analysis of gelatin and GelMA. **(B)** SEM images of cross-sections for GelMA, 100-GO/GelMA and 200-GO/GelMA hydrogels at low (150×) and high (×500) magnifications, demonstrating their interconnected porous architecture, with no marked alteration in pore structure following GO incorporation.

**FIGURE 4:**
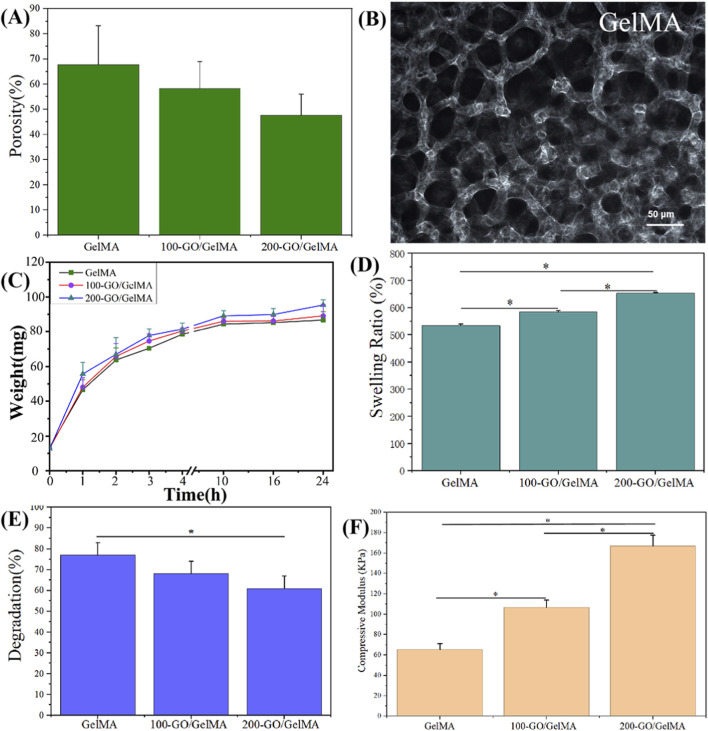
Characterization of GO/GelMA hydrogels. **(A)** Porosity of the hydrogels, showing a gradual decrease with increasing GO concentration. **(B)** Light microscope images of GelMA hydrogel after 24 h of swelling, highlighting the porous structure with interconnected pores. **(C)** Weight change kinetics of hydrogels immersed in PBS over 24 h. **(D)** Swelling behevior of the hydrogels, demonstrating a concentration-dependent increase in swelling ratio. **(E)** Degradation rates of the hydrogels after 4 weeks of immersion in PBS, indicating a significant reduction in degradation with higher GO concentrations. **(F)** Compressive modulus of the hydrogels, showing significant increases in elastic modulus with increasing GO content. *P < 0.05.

The HNMR results of gelatin and GelMA are shown in Figure 3A. In the spectrum of gelatin, the methylene-lysine signal at 2.9 ppm was nearly absent. In the spectrum of GelMA, new signals appeared at 5.4 ppm and 5.6 ppm, corresponding to the methacrylate groups. These changes indicate that methacrylic anhydride was successfully grafted onto the lysine residues of gelatin. According to the calculation Formula 1, the degree of substitution in this experiment was 87%, confirming the successful synthesis of GelMA with a high substitution rate. SEM images of hydrogel cross-sections (Figure 3B) showed that the pore structure was maintained after GO incorporation, although porosity decreased gradually with increasing GO concentration (GelMA: 68%, 100-GO/GelMA: 58%, 200-GO/GelMA: 48%; Figure 4A). The differences were not statistically significant (*P > 0.05*, Figure 4A). Figure 4B shows the light microscopy image of the GelMA hydrogel after 24 h of water absorption, displaying a porous structure with interconnected pores of varying sizes (Figure 4B).

The swelling kinetics, presented as weight change over time, are shown in Figure 4C. All hydrogels absorbed PBS rapidly within the first 6 h, followed by a gradual approach to equilibrium swelling. The swelling ratios after 24 h of immersion in PBS were 540% ± 6.3% (GelMA), 596% ± 4.5% (100-GO/GelMA), and 655% ± 3.8% (200-GO/GelMA), with significant differences among the groups (Figure 4D, *P < 0.05*). After 4 weeks, the degradation rate were 77% ± 6.1%, 68% ± 6%, and 61% ± 6.1%, respectively, indicating that GO incorporation significantly slowed degradation (Figure 4E, *P < 0.05*). The compressive elastic modulus of the GelMA group was 65.3 ± 5.7 kPa, that of the 100-GO/GelMA group was 106.7 ± 7.1 kPa, and that of the 200-GO/GelMA group was 167 ± 10.6 kPa, representing increases of 63% and 156% compared to the GelMA group, respectively (Figure 4F, *P < 0.05*).

### 3.2 Antibacterial performance of GO/GelMA

**FIGURE 5:**
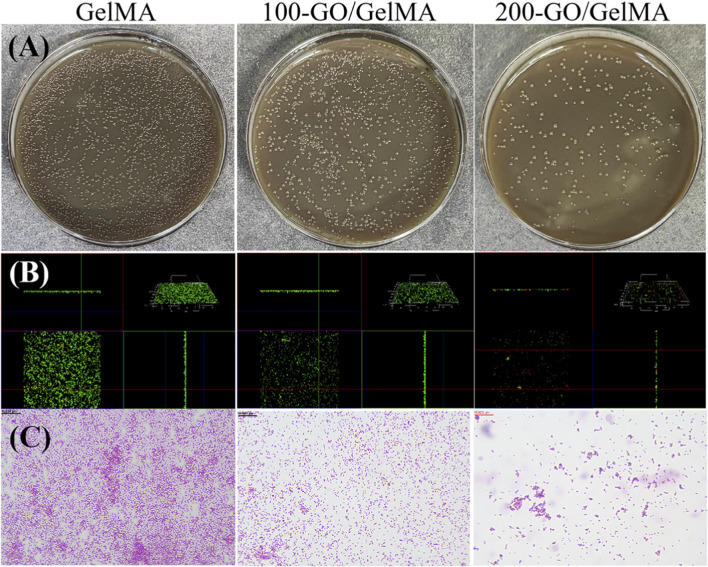
Evaluation of the antibacterial performance of GO/GelMA hydrogels against *Porphyromonas gingivalis*. **(A)** Colonization analysis of bacterial survival rates, showing a concentration-dependent decrease with GO incorporation. **(B)** Live/Dead bacterial staining images (green: live bacteria; red: dead bacteria), confirming enhanced bactericidal activity. **(C)** Gram staining results, indicating a reduced bacterial load in GO-containing groups.

The antibacterial experiment demonstrated that the addition of GO significantly enhanced the antibacterial properties of the hydrogels, with bacterial survival rates decreasing as GO concentration increased (Figure 5A). Live/dead bacterial staining (Figure 5B) and Gram staining results (Figure 5C) confirmed the highest bacterial load in the pure GelMA group and the lowest in the 200-GO/GelMA group.

### 3.3 BMSC viability

**FIGURE 6:**
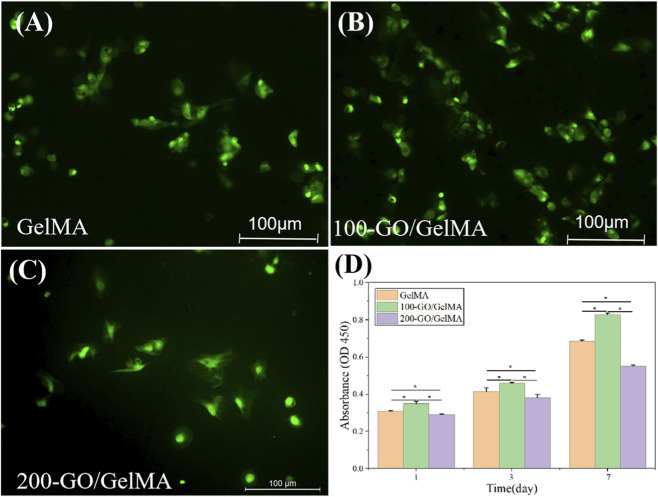
Biocompatibility and proliferation of BMSCs on GO/GelMA hydrogels. **(A–C)** Cell viability of BMSCs after 3 days of culture, showing high cell viability across all groups. **(D)** CCK-8 assay quantifying BMSC proliferation over 7 days, indicating that the 100-GO/GelMA group supported the highest cell viability. *P < 0.05.

Live/dead staining results showed that after 3 days of culture, the density of live cells in the 100-GO/GelMA group was significantly higher than in the other groups (Figures 6A–C). CCK-8 assay results showed that cell viability increased initially and then decreased with increasing GO concentration. The viability of BMSCs in the 100-GO/GelMA group was significantly higher than in the other two groups on days 1, 3, and 7 (Figure 6D, *P < 0.05*).

### 3.4 *In vitro* osteogenic performance

**FIGURE 7:**
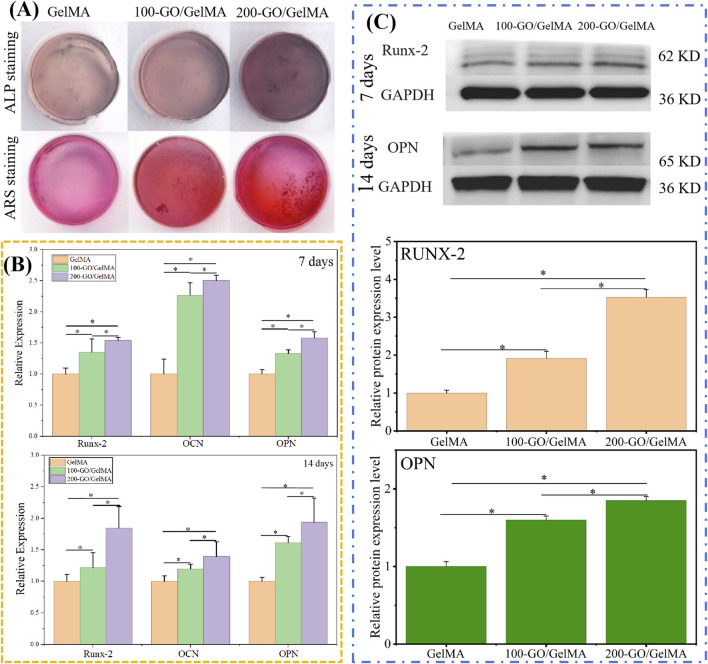
*In vitro* osteogenic differentiation of BMSCs on GO/GelMA hydrogels. **(A)** ALP and ARS staining, demonstrating enhanced early and late osteogenic differentiation in a GO-concentration-dependent manner. **(B)** RT-qPCR analysis of osteogenic gene expression (Runx2, OCN, OPN) after 7 and 14 days of osteogenic induction, showing significant upregulation in GO groups. **(C)** WB analysis confirming the increased expression of osteogenic proteins (Runx2, OPN) at both time points. *P < 0.05.

As is shown in Figure 7A, ALP staining results showed that as GO concentration increased, the color intensity of ALP staining deepened, indicating that GO effectively promoted the osteogenic differentiation of BMSCs. ARS results also showed that calcium deposition increased significantly with increasing GO concentration, further confirming the osteogenic properties of GO.

RT-qPCR (Figure 7B) showed that the expression levels of osteogenesis-related genes (Runx-2, OCN, and OPN) in the 100-GO/GelMA and 200-GO/GelMA groups were significantly higher than in the GelMA group, and the 200-GO/GelMA group was significantly higher than the 100-GO/GelMA group, indicating that the addition of GO effectively promoted the osteogenic differentiation of BMSCs. In addition, we observed upregulated protein levels of Runx-2 and OPN in groups 100-GO/GelMA and 200-GO/GelMA, with Group 200-GO/GelMA showing the highest expression level (Figure 7C). Densitometric analysis revealed that the 200-GO/GelMA group showed the highest expression, with Runx2 and OPN levels approximately 3.5-fold and 1.8-fold higher than those in the GelMA control, respectively.

### 3.5 *In vivo* anti-inflammatory and osteogenic effects

**FIGURE 8:**
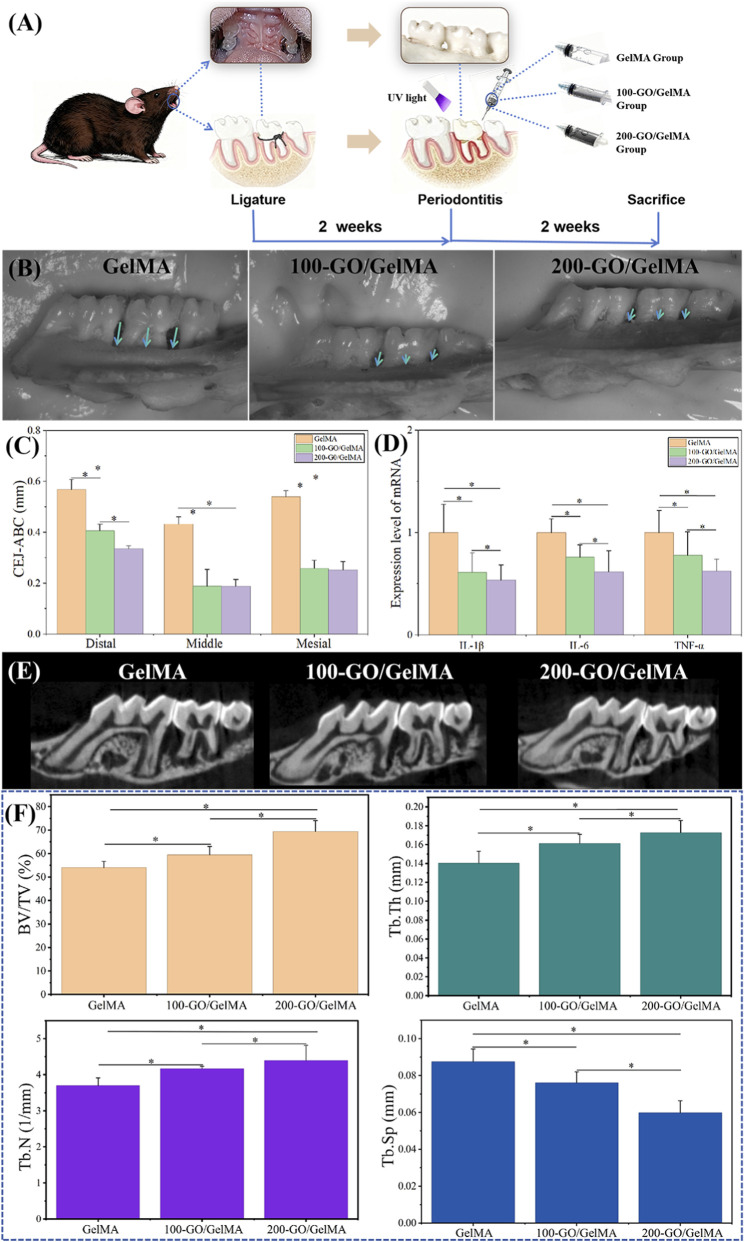
*In vivo* evaluation of anti-inflammatory and osteogenic effects in a periodontitis mouse model. **(A)** Schematic diagram of the experimental timeline and procedures. Stereomicroscope images **(B)** and quantitative measurement **(C)** of CEJ-ABC distance, indicating that the 200-GO/GelMA group most effectively inhibited alveolar bone loss. **(D)** RT-qPCR analysis of pro-inflammatory cytokine (IL-1β, IL-6, TNF-α) expression in gingival tissues, confirming the anti-inflammatory effect of GO/GelMA hydrogels. **(E)** Representative 3D reconstructed Micro-CT images of the alveolar bone and **(F)** quantitative analysis of bone morphometric parameters (BV/TV, Tb.N, Tb.Th, Tb.Sp). *P < 0.05*.

In the periodontitis model, the therapeutic effects of multifunctional GO/GelMA on periodontitis were investigated, as shown in Figure 8A. The stereomicroscopic observation results showed that after 2 weeks of hydrogel treatment, the CEJ-ABC values in the 200-GO/GelMA group were significantly lower than those in the other two groups, and the values in the 100-GO/GelMA group were also significantly lower than those in the GelMA group (Figures 8B,C, *P < 0.05*). The expression of pro-inflammatory cytokines (IL-6, IL-1β and TNF-α) in the gingival tissues from the 200-GO/GelMA group and 100-GO/GelMA group were significantly lower than that observed in the GelMA group (Figure 8D, *P < 0.05*). Micro-CT 3D reconstruction (Figure 8E) and quantitative analysis (Figure 8F) confirmed that the 200-GO/GelMA treatment resulted in the most significant bone regeneration, evidenced by the highest BV/TV and Tb.N, and the most favorable Tb.Th and Tb.Sp values.

## 4 Discussion

Tissue engineering offers excellent opportunities for effectively repairing bone defects caused by periodontitis, with biocompatible scaffolds being the most critical factor. This study developed an integrated GO/GelMA hydrogel that simultaneously addresses infection control and bone regeneration in periodontitis. The key finding is that 200 µg/mL GO represents an optimal concentration, balancing effective antibacterial activity with potent osteoinductive capacity, despite a mild initial cytostatic effect on cell proliferation.

The improved mechanical properties and modulated degradation profile are advantageous for a bone graft scaffold, providing initial stability and space maintenance. Although GO addition reduced porosity, the values remained within the range of cancellous bone porosity (30%–90%), which is crucial for cell adhesion and nutrient transport (Bose et al., 2012; Bose et al., 2013). The swelling behavior of the GO/GelMA hydrogels showed a significant concentration dependence, with the 200-GO/GelMA group was the highest, while the degradation rate was the lowest. This may be related to the hydroxyl groups (OH) in GO, which enhance hydrophilicity and reduce degradation rates. This hydrophilicity is important for maintaining cell activity and promoting cell proliferation (Saravanan et al., 2018).

In terms of antibacterial performance, this study verified the significant antibacterial effect of GO/GelMA hydrogels on periodontitis-related bacteria through *in vitro* experiments. The bacterial survival rates and total bacterial counts of the 100-GO/GelMA and 200-GO/GelMA groups were significantly lower than those of the control group (P < 0.05), indicating that the introduction of GO effectively enhanced the antibacterial properties of the hydrogels. The mechanism may be related to the damage of bacterial cell membranes by GO and the oxidative stress induced (Pumera, 2010; Liu et al., 2011).

However, this mechanism is mainly observed when GO is in suspension state. When GO was homogeneously distributed in polymer matrix, the GO interface control and dispersion are altered, reducing its capacity for the mechanical damage and hence the antibacterial properties (Aslam Khan et al., 2020). This is likely because when GO is uniformly dispersed within the GelMA polymer matrix, its sharp edges become encapsulated, reducing direct physical contact with bacteria and thereby weakening the key antibacterial mechanism of physical piercing and disruption of cell membranes. Consequently, a higher concentration of GO is required to ensure that a sufficient number of nanosheets are exposed on the hydrogel surface or released, enabling an effective antibacterial effect. Therefore, the higher antibacterial concentration required in this study compared to some literature reports (He et al., 2015) does not represent a contradiction, but rather reflects a shift in the mechanism of action and effective concentration of GO within this specific material system (the GelMA hydrogel). This highlights that when applying GO, the interaction between GO and the carrier material must be considered, and the concentration of GO itself cannot be viewed in isolation.

Regarding osteogenic performance, this study systematically evaluated the ability of GO/GelMA hydrogels to promote the osteogenic differentiation of BMSCs. The results showed that the viability of BMSCs in the 100-GO/GelMA group was significantly higher than in the other groups at early stages. However, from day 3 onwards, the viability in the 200-GO/GelMA group was lower, indicating that high concentrations of GO (≥200 µg/mL) may induce cytostatic effects or promote cell death. Kim et al. (2013) found that when GO was incorporated into chitosan, no cytotoxicity was observed at concentrations <100 µg/mL, but cytotoxicity was observed at concentrations >100 µg/mL, which is consistent with our results. Zhang et al. (2015) explained that as the concentration of GO increases, the oxygen-containing functional groups on its surface induce reactive oxygen species (ROS) in cells, thereby inhibiting cell proliferation.

Notably, despite the initial suppression of proliferation, the 200-GO/GelMA group demonstrated the most potent osteogenic differentiation, as evidenced by ALP activity, calcium deposition, and the expression of osteogenic genes and proteins. The reasons maybe that the higher concentration of GO, with its abundant surface oxygen-containing functional groups (e.g., -COOH, -OH), may adsorb higher concentrations of osteogenesis-related proteins (such as fibronectin, bone morphogenetic proteins) and calcium/phosphate ions, creating a more inductive microenvironment around the cells (Zhang et al., 2021). Therefore, at the 200 µg/mL concentration, the potent biochemical inductive signals provided by GO may surpass its mild inhibitory effect on cell proliferation, ultimately leading to a more pronounced osteogenic differentiation outcome. This is consistent with the results of previous studies (Noh et al., 2017; Saravanan et al., 2018). For example, Saravanan et al. (2018) prepared a chitosan-based thermoresponsive hydrogel containing GO and found that GO exhibited good osteogenic differentiation of BMSCs; Noh et al. (2017) found that GO-functionalized PEGDA hydrogels (PEGDA-GO) enhanced the osteogenic differentiation of stem cells compared to PEGDA alone under osteogenic induction conditions. These studies indicate that the addition of GO within a certain concentration range can significantly improve the osteogenic properties of hydrogels, making them potentially valuable for the treatment of periodontitis-induced bone defects.

Similar to the *in vitro* results, periodontitis model mice treated with the hydrogel for 2 weeks also showed the least alveolar bone loss in the 200-GO/GelMA group, which was significantly better than that in the other two groups. The levels of gingival inflammatory factors in the 200-GO/GelMA group were also significantly lower than those in the other two groups.These results confirm that this concentration provides the composite hydrogel with the best overall performance in terms of both anti-inflammatory and osteogenic properties.

### 4.1 Limitations and future perspectives

This study has several limitations. First, the optimal concentration of GO remains to be further explored, and the long-term biosafety and potential chronic immune response to the degradation products of GO *in vivo* require further investigation. Second, the antibacterial efficacy should be validated against polymicrobial biofilms rather than a single species (*P. gingivalis*). Future research should focus on addressing these limitations to fully realize the potential of GO/GelMA hydrogels for treating periodontitis-induced bone defects.

## 5 Conclusion

In summary, the photocrosslinked GO/GelMA composite hydrogel, particularly at a GO concentration of 200 µg/mL, synergistically combines strong antibacterial activity against *P. gingivalis* with potent osteoinductive properties. It also exhibits suitable physical characteristics for bone tissue engineering. This multifunctional scaffold presents a highly promising strategy for regenerating bone defects in periodontitis and warrants further translational development.

## Data Availability

The original contributions presented in the study are included in the article/Supplementary Material, further inquiries can be directed to the corresponding authors.
