# Supplementary material for: Synergistic antibacterial and osteogenic activities of GO/GelMA hydrogel for regenerating periodontal bone defects
Source: Front Bioeng Biotechnol. 2026 Apr 23;14:1744639. doi: 10.3389/fbioe.2026.1744639 (PMC13149429; doi:10.3389/fbioe.2026.1744639)
Supplement: Supplementary file 1 [file Table1.docx]

Synergistic Antibacterial and Osteogenic Activities of GO/GelMA Hydrogel for Regenerating Periodontal Bone Defects

Cheng Cheng^1^， Guangna Yue^2,*^，Lu Qiang^1,*^

^1^Department of Stomatology, Beijing University of Chinese Medicine Third Affiliated Hospital, Beijing 100029, China

^2^Department of Stomatology, Shanghai East Hospital, Tongji University School of Medicine, Shanghai 200120, China

*Corresponding author: Guangna Yue(guangnayue@sina.com); Lu Qiang(957596499@qq.com)

Table S1 The primers for RT-PCR used in the present study

| Gene name | Primer sequence (5’→3’) | Species |
| --- | --- | --- |
| Runx2 | Forward:CTGAGAGGGAAATCGTGCGTGAC | rat |
|  | Reverse:AGGAAGAGGATGCGGCAGTGG |  |
| OCN | Forward:GGTGCAGACCTAGCAGACACCA | rat |
|  | Reverse:AGGTAGCGCCGGAGTCTATTCA |  |
| OPN | Forward:CAGTCGATGTCCCTGACGG | rat |
|  | Reverse:GTTGCTGTCCTGATCAGAGG |  |
| IL-1β | Forward:5′-CACCTTTTGACAGTGATGAG-3′ | mouse |
|  | Reverse:5′-AGCCACAATGAGTGATACTG-3′ |  |
| IL-6 | Forward:5′-ACTTCACAAGTCGGAGGCTT-3′ | mouse |
|  | Reverse:5′-TGCAAGTGCAT CATCGTTGT-3' |  |
| TNF-α | Forward:5′-ATGAGCACAGAAAGCATGATC-3′ | mouse |
|  | Reverse:5′-TACAGGCTTGT CACTCGAATT-3' |  |
